# Editorial: The new frontier in brain network physiology: from temporal dynamics to the principles of integration in physiological brain networks

**DOI:** 10.3389/fncom.2023.1242834

**Published:** 2023-07-03

**Authors:** Carlos Trenado, Ignacio Mendez-Balbuena, Alena Damborská, Amir Hussain, Mufti Mahmud, Mohammad Reza Daliri

**Affiliations:** ^1^Institute of Clinical Neuroscience and Medical Psychology, Medical Faculty, Heinrich Heine University, Duesseldorf, Germany; ^2^Facultad de Psicología, Benemérita Universidad Autónoma de Puebla, Puebla, Mexico; ^3^Central European Institute of Technology (CEITEC), Brain and Mind Research Program, Masaryk University, Brno, Czechia; ^4^Department of Psychiatry, Faculty of Medicine, University Hospital Brno, Masaryk University, Brno, Czechia; ^5^School of Computing, Edinburgh Napier University, Edinburgh, United Kingdom; ^6^Department of Computer Science, Nottingham Trent University, Nottingham, United Kingdom; ^7^Neuroscience and Neuroengineering Research Laboratory, Biomedical Engineering Department, School of Electrical Engineering, Iran University of Science and Technology, Tehran, Iran

**Keywords:** network, temporal, physiology, integration, brain network, dynamics, variability

The outstanding processing capacity of the human brain not only enables us to respond to a complex environment and survive, but also gives rise to memorable and pleasurable experiences, such as the aesthetic perception of music, arts and nature. To do so, the brain integrates information from a vast number of neurons organized into brain structures and more or less specialized large-scale brain networks to achieve its functionality. In particular, long-range communication between neural assemblies takes place to facilitate the representation, storage and retrieval of information in neural circuits as well as to process and react to the plethora of external stimuli present in the environment. The mechanism to achieve such an intricate task remains a key issue in human neurobiology and a source of ideas and progress in the field of brain network physiology.

One of the most distinctive patterns of neural activity that emerges over time regardless of the considered spatial scale (neurons, neural assemblies, brain structures, topographic maps, organs of the central nervous system) is the occurrence of oscillatory rhythms. Brain oscillations may result from the synchronized activity of neurons due to processing of a particular task or represent only a putative mechanism resulting from the interaction of neurons and brain structures. With regard to the first, it has been suggested that synchronization of neuronal activity at different spatial and temporal scales mediates brain communication (Schnitzler and Gross, 2005; Sejnowski and Paulsen, 2006). Other relevant characteristic of neuronal activity is the reduction of variability in neural response and habituation upon presentation of the same stimuli, which is apparent in the case of oscillatory activity (Daniel et al., 2019). In fact, such neural variability could represent a mechanism of neural redundancy that ensures proper transmission of information in the brain while also providing information on altered brain states as in the case of patients with neurological and psychiatric diseases (Trenado et al., 2019).

It is timely to emphasize that the study of the human brain has relied on outstanding advances in the integration of physiological measurements, neuroimaging techniques, brain stimulation, and computational modeling approaches that undoubtedly have made us progress toward a better understanding of brain capacity and important features that make us unique as humans such as empathy, creativity and resilience. In the next ten years, we expect to see great progress in understanding brain mechanisms in situations beyond the traditional laboratory setting, namely by considering the complexity of realistic environments and conditions that are present in our daily life, including clinical settings. Indeed, such efforts will require the adoption of novel technologies such as virtual reality and artificial intelligence in combination with traditional methodologies as well as a multidisciplinary approach to tackle the most pressing issues that will take us to the next level and improve the quality of life of humans all over the planet.

The research articles in this Research Topic represent examples of innovative views and ideas toward the relevant issue of cognition and perception in humans. The techniques used reflect the rich interplay between different disciplines and views in pursuing a deeper understanding of the human brain cognitive mechanisms in relation to physiology at different level of detail. In what follows, we discuss the included works.

Human sensory perception in a virtual reality environment (VRE) is an emerging topic as emphasized by disciplines such as education, entertainment and medical applications. In particular, the aspect of altered motion perception in a VR environment is of high importance as it influences comfortability of a user as well as the ecological validity of such an environment. To address this, Ahn et al. studied the temporal dynamics in VRE and their attributed effect on discomfort, nausea or headache, due to visual vestibular conflicts. They speculated about an altered self-motion perception by accelerative visual motion stimulation due to the absence of vestibular signals (visual-vestibular sensory conflict) in the VRE. The results indicated changes in neural dynamics under the sensory mismatch condition (accelerative visual motion stimulation), particularly in participants with high levels of motion sickness under driving simulation. An event-related potential study revealed higher P3 amplitudes in those who experienced high motion sickness, as attributed to a substantial demand of cognitive resources for motion perception on sensory mismatch conditions (Ahn et al.).

Music is not only and array of sounds to create some combination of harmony, melody or expressive content, but also a powerful stimulus that may affect the relationship between mind and body. In this respect, Dell'Anna et al. addressed the interesting aspect of music interaction and its effect on embodiment. The authors suggested that the proper way to capture the socially interactive nature of music or musicality is to conceive it as an embodied language, rooted in culturally adapted brain structures. They propose a framework of music as an embodied language and discuss its implications in three experimental studies. The framework then establishes a link between cognitive musicology and neuroscience. That is, social neuroscience deals with the neural underpinnings of human interaction as mediated by language. By considering music and musicology as an embodiment of language, facilitation of such interaction may occur with outstanding affective and esthetic consequences (Dell'Anna et al.).

Pitch refers to frequency characteristics that are present in a sound, melody or speech's prosody. Given the involvement of different auditory neurons and cells in shaping the properties of pitch perception, the use of computational models appears suitable to understand the effect of different network configuration parameters such as neural connectivity as well as periodicity properties of auditory input stimuli on auditory processing and perception. In this regard, Feldhoff et al. have used a biologically inspired computational model of the auditory pathway to explain the perception of pitch's periodicity, which is characteristic of musical instruments and sung vowels. In the present work, they were able to incorporate the effect of octopus cells, which are tonotopically arranged as part of the posteroventral cochlear nucleus and receive input from auditory-nerve fibers. Based on the simulation results, the authors reproduced the behavior of an octopus cell with regard to pitch discrimination, estimation and evaluation (Feldhoff et al.).

A dual-task (DT) is an experimental neuropsychology test that requires an individual to perform two tasks simultaneously. DT involves coordination of executive function and attention between a learning task and an additional task intended to capture the individual's cognitive capacities during the learning process. The aspect of interference between both tasks is relevant for assessing the capacity of an individual to perform tasks simultaneously. In particular, Sadeghi Talarposhti et al. addressed the interference behavioral effect that occurs in a DT both experimentally and by using computational modeling. The authors proposed a black-box neural model based on neurological studies and oscillator units to represent neural activities. Comparison between the model's output and experimental results validated the proposed approach while encouraging its use in studying other DT paradigms (Sadeghi Talarposhti et al.).

Hazani focused on the important topic of cellular evolution by providing arguments in support of the hypothesis that cell life evolves toward increasing an organism's resonant energy transfer or “exposing points” with its natural environment.

This Research Topic has presented the new field of brain network physiology, which investigates how different physiological systems and sub-systems of the brain interact and combine their dynamics to produce various functions and states at the organism level. Even so, the papers in this Research Topic have investigated some of the main concepts and methods and some of the current challenges and open questions of this field but the field is growing fast and we hope to see more papers in this direction to uncover novel insights into the mechanisms of physiological brain networks.

## Author contributions

All authors listed have made a substantial, direct, and intellectual contribution to the work and approved it for publication.
